# Health Benefits and Applications of Goji Berries in Functional Food Products Development: A Review

**DOI:** 10.3390/antiox11020248

**Published:** 2022-01-27

**Authors:** Bojana B. Vidović, Danijel D. Milinčić, Mirjana D. Marčetić, Jelena D. Djuriš, Tijana D. Ilić, Aleksandar Ž. Kostić, Mirjana B. Pešić

**Affiliations:** 1 Department of Bromatology, Faculty of Pharmacy, University of Belgrade, Vojvode Stepe 450, 11221 Belgrade, Serbia; tijana.ilic@pharmacy.bg.ac.rs; 2 Department of Chemistry and Biochemistry, Faculty of Agriculture, University of Belgrade, Nemanjina 6, 11080 Belgrade, Serbia; danijel.milincic@agrif.bg.ac.rs (D.D.M.); akostic@agrif.bg.ac.rs (A.Ž.K.); mpesic@agrif.bg.ac.rs (M.B.P.); 3 Department of Pharmacognosy, Faculty of Pharmacy, University of Belgrade, Vojvode Stepe 450, 11221 Belgrade, Serbia; mirjana.marcetic@pharmacy.bg.ac.rs; 4 Department of Pharmaceutical Technology and Cosmetology, Faculty of Pharmacy, University of Belgrade, Vojvode Stepe 450, 11221 Belgrade, Serbia; jelena.djuris@pharmacy.bg.ac.rs

**Keywords:** goji, bioactive compounds, antioxidant properties, health benefits, processing, food product development

## Abstract

Goji berries have long been used for their nutritional value and medicinal purposes in Asian countries. In the last two decades, goji berries have become popular around the world and are consumed as a functional food due to wide-range bioactive compounds with health-promoting properties. In addition, they are gaining increased research attention as a source of functional ingredients with potential industrial applications. This review focuses on the antioxidant properties of goji berries, scientific evidence on their health effects based on human interventional studies, safety concerns, goji berry processing technologies, and applications of goji berry-based ingredients in developing functional food products.

## 1. Introduction

Berry fruits are frequently consumed worldwide due to their richness in highly valuable bioactive compounds, which potentially positively impact human health [1,2]. In addition to dietary fibers, vitamins, and minerals, berries contain phytochemicals, such as phenolic compounds and carotenoids, which exert antioxidant, anti-inflammatory, and many other health-promoting effects [3]. Berries are consumed fresh, frozen, or dried and used as ingredients in different food products and dietary supplements [4]. As a marketing strategy to promote their extraordinary health benefits, berries are widely advertised as superfruits [5] and functional foods [6]. Among exotic berry fruits, goji berries are gaining more importance in different countries, both from medical and pharmaceutical standpoints, as well as their further application in the food industry.

The genus *Lycium* (Solanaceae) comprises about 100 species distributed from temperate to subtropical regions [7]. The fruits known as goji berries, wolfberries, barbary wolfberry, and Chinese boxthorn (or *gouqizi* in Chinese) might derive from two closely related species, *Lycium barbarum* L. and *L. chinense* Mill. *Lycium barbarum* is a perennial deciduous shrub with ellipsoid orange–red berries and a sweet–tangy flavour. The original area is not definitively established, but it could be between Southeast Europe and Southwest Asia. The plant is widely distributed in warm regions, particularly in the Mediterranean, Southwest, and Central Asia. *Lycium chinense* is native to China, Taiwan, and Japan, and is widely cultivated in Asia, but is also naturalized in Europe and the United States. The black goji fruits with a specific composition and taste are obtained from Chinese native species *L. ruthenicum* Murr. [8,9,10].

The first reports about goji berries are associated with their use in traditional Chinese medicine in the form of mild Yin tonics, tinctures, and powders [11]. In addition, since ancient times, these berries have been highly valued and used in raw, dried, or processed forms, such as tea, juice, wine, or liqueur [12]. Due to effective instrumental techniques, the complex composition of goji berries has been examined in detail in recent years. It has been shown that goji berries are a good source of nutrients such as lipids, proteins, fibres, vitamin C, and minerals [13,14], and non-nutritive bioactive compounds such as phenolic compounds, polysaccharides, and carotenoids [15,16]. On the other hand, in vitro antioxidant assays, in vivo studies, and clinical trials have contributed to understanding some of the health benefits of goji berries [8,16,17,18]. This knowledge has created a new concept in diets, aiming to develop and promote goji berries as a functional food, or their use in formulating innovative food products. For these reasons, at the beginning of the twenty-first century, goji berry cultivation spread outside China and other Asian countries. Precisely, there are reports that goji berries are cultivated throughout Europe, including in Italy [17,19], Portugal [20], Greece [15,21,22], Romania [16], Bulgaria [23], Serbia [24,25], and North Macedonia [26], as well as Slovenia [2], Switzerland [27], Poland [28], and Lithuania [29]. Despite observed variations in chemical compositions and bioactivities among goji berries from different regions, affected by genotypes, environmental conditions, and many other pre-harvest and post-harvest factors, the specific profile of primary and secondary metabolites and good antioxidant properties makes goji berries convenient for further applications in the pharmaceutical and food industries. Today, goji berries are available as food or food supplements on the global functional food market. In Italy, goji fruit has been added by the Ministry of Health to the list of foods with physiological antioxidant properties, and is commonly found in food supplements. Goji berries are also present in various food products, including in ice-creams, marmalades, sauces, salads, and beer, as well as bakery and dairy products [30]. Although, in general, the goji berry has no long history of significant consumption in Europe, the goji berry is not regulated under the EU novel foods legislation, and has no restrictions or specific legislation requirements for its use and further food applications. Among different identified safety concerns, there is growing evidence that goji berries may trigger allergic reactions in sensitive individuals, especially across Mediterranean populations [31,32].

## 2. Nutritional Value and Bioactive Compounds of Goji Berries

**Figure 1:**
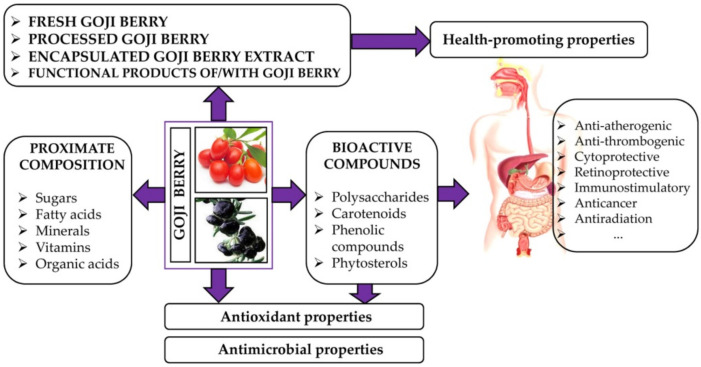
Biological activities of goji berry and its products.

Specific colours, from yellow and red (*L. barbarum*) to black (*L. ruthenicum*), as well as a combined sweet, tangy, and pungent taste, make goji berries very attractive to consumers [25]. The balanced content of sugars (fructose, glucose, and sucrose), organic acids, and specific secondary metabolites is responsible for the acceptable sensory characteristics of raw and dried goji berries, as well as for the refreshing character of different food products enriched with them [12,13,18] (Figure 1). In the nutritional term, goji berries present a source of dietary fibres [13,14,25], vitamin C [8,14,24,33,34], and some minerals, including potassium, copper, manganese, iron, and zinc [13,14,25,26,35]. In addition, microelements from *L. barbarum* fruits have high bioaccessibility [36]. The primary fatty acids of goji berries are linoleic acid, followed by oleic, palmitic, and stearic acids (about 95% of the total fatty acids) [20,25,26,33,37]. The most abundant amino acids in goji berries are proline and serine, while the essential amino acids represent up to 30% of total free amino acids [34]. In addition, goji berries have characterized non-protein amino acids, such as *γ*-aminobutyric acid, hydroxyproline, and citrulline, with specific metabolic functions [38].

Goji berries exert various biological activities and health benefits, such as antioxidant, anti-inflammatory, antimicrobial, immuno-stimulating, anti-diabetic, neuroprotective, anti-cancer, prebiotic, and anti-obesogenic effects, which have been reviewed by several authors [9,18,39,40,41] (Figure 1). These beneficial properties are attributed to the individual or combined effects of the constituents of goji berries [18,42]. Water-soluble polysaccharides (*L. barbarum* polysaccharides, LBPs) are considered to be the most important bioactive components of goji berries [12,43]. In addition to pectic polysaccharides, as major compounds, LBPs are composed of glucan, xylan, and arabinogalactan-proteins [44]. The main chains of the glycan backbones of LBPs are composed of (1→3)-*β*-D-galactopyranosyl, (1→6)-*β*-D-galactopyranosyl, and (1→4)-*α*-D-galactopyranosyl-uronic acid residues [45,46]. The LBPs account for 5–8% of the dried fruit [12]. In fact, the yield of LBPs was proposed as a parameter to evaluate the quality of *L. barbarum* and its applicability for medicinal and functional food use [47]. However, structural features including molecular weight, type, and the ratio of monosaccharides, glycosidic linkage patterns, and chain conformations may strongly affect LBPs bioactivities [45,46,48]. Based on the investigation of the structure–bioactivity relationship, it is assumed that the immunomodulation effects of LBPs originate from different partial acid and enzymatically hydrolysed fragments [45,46,49]. Animal model studies have shown that the oral administration of LBPs (5, 10, and 20 mg/kg/day) [50] or goji berry extract [51] improves food conversion rate, reduces body weight, and diminishes insulin resistance. Moreover, in vivo mouse model studies have shown that LBPs at a dose of 0.1 mL/10 g body weight modulate the immune response and affect the intestinal microbiota, stimulating the growth of some probiotic genera [40]. Goji berries are a good source of phenolic compounds, including phenolic acids, flavonoids, phenylpropanoids, coumarins, lignans, and their derivatives [8,18,20,51], which selectively contribute to their bioactivities [51]. Several studies have shown that the phenolic extracts of goji berries exert good in vitro antioxidant activities and antimicrobial effects against some Gram-negative and Gram-positive bacteria [20,25,52]. In addition to antioxidative properties, *L. ruthenicum* anthocyanins have demonstrated anti-inflammatory [53,54], antilipidemic [55], and antiobesity properties [56]. While these water-soluble flavonoids are responsible for the purplish–blue colour of black goji berry, high carotenoid content, predominant zeaxanthin, and its esters result in the red–orange colour of red goji berries [57]. Moreover, *L. barbarum* berries contain a higher zeaxanthin content than other zeaxanthin-rich foods, such as egg yolks [58]. Since it accumulates in the retina, the protective effects of goji berry against age-related macular degeneration and cataracts are attributed to the presence of zeaxanthin and its antioxidant properties [12]. Furthermore, goji berries contain monoterpenes (phellandrene, sabinene, terpinene) and vitamins [8]. Moreover, goji berries contain 2-*O*-*β*-D-glucopyranosyl-L-ascorbic acid (AA-2*β*G), which hydrolyses via *α*-glucosidase in the intestinal tract to active L-ascorbic acid [59].

However, the nutritional composition, bioactive compound profiles, and biological properties of goji berries largely depend on genotypes, affecting their further applications. For example, while *L. barbarum* berries had a predominant total carotenoid and AA-2βG, *L. barbarum* var. *auranticarpum* (a yellow fruit variety) had high levels of flavonoids and pronounced antimicrobial properties; then, *L. ruthenicum* berries extract had the highest total phenolic content, and the best antioxidant activity [25]. In addition to genotypic differences [16,25,60], bioactive compounds in goji berries are affected by geographic origin [17,38], harvesting time [26,61], and post-harvesting factors [62]. Table 1 summarises recently reported data on the total phenolics, flavonoids, carotenoid and polysaccharides content in red and black goji berries.

## 3. Antioxidant Properties of Goji Berries

The goji berry is unique in its types and its overall content of bioactive compounds [5]. Compared to other common fruits, goji berry presented less antioxidant capacity than blackcurrant and blueberry, but more than kiwifruit, raspberry, and orange [8]. In addition, several studies indicate that black goji berries have more potent antioxidant properties than red goji berries [25,63,66,70] (Figure 1). The antioxidant activities of goji berries are closely associated with the presence of polysaccharides, carotenoids, flavonoids, and AA-2βG [71]. These compounds can exhibit antioxidant effects in several ways: radical scavenging activities toward reactive species via hydrogen atom transfer or electron donation, through metal chelation, or interactions with other antioxidants [72].

The antioxidant effect of flavonoids is influenced by the number of hydroxyl groups on the B ring of their structure. Flavonoids with two hydroxyl groups in the B ring with *ortho* arrangement had higher scavenging activity. Moreover, due to higher molecule flexibility, flavonoids with single bonds between C2 and C3 showed better antioxidant ability than structures with double bonds. In addition to radical scavenging activities, flavonoids exhibit metal ion chelating and reducing ability [20,72]. Anthocyanins donate hydrogen atoms to highly reactive free radicals and block free radical chain reactions. Similar to other flavonoids, the scavenging activity of anthocyanins correlates with the number of hydroxyl groups. Different phenolic compounds in goji berry can have synergistic, additive, or rarely antagonistic antioxidant effects. These interactions are usually concentration-dependent, and are likely due to how phenolic compounds interact with different free radicals. In addition, flavonoids and other phenolics could increase the expression of antioxidant enzymes, such as catalase, glutathione peroxidase, and superoxide dismutase (SOD), and suppress the formation of reactive oxygen species [9,18,19,69]. Due to the highest phenolic content, the pulp of goji berries has the highest contribution rate to antioxidant capacities compared to seeds and whole fruits [66].

Goji polysaccharides (LBPs) exhibited anti-lipid peroxidation activity, reducing capacity and radical scavenging activity towards the superoxide anion. The activity was similar to those of the synthetic antioxidant, butylated hydroxytoluene (BHT) [9]. Lin et al. (2009) evaluated the antioxidant activity of neutral polysaccharides (LBPN) and three acidic polysaccharides isolated from goji berry, and compared with crude polysaccharide (CP), crude extract of polysaccharide (CE), deproteinated polysaccharide (DP), and deproteinated and dialyzed polysaccharide (DDP). Except for CE and DDP, most polysaccharide fractions at high concentrations exert effective scavenging radical activities. DDP and CE demonstrated lower reducing power, while LBPN and CE showed poor metal ion chelating activity compared to other polysaccharide fractions [73]. Similarly, Wang et al. (2010) demonstrated a poor ferrous ion-chelating effect for LBPN and CE, whereas moderate chelating activities were noticed for CP and acidic polysaccharides [74]. The ability of the carboxyl group from galacturonic acid to scavenge radicals and chelate metal ions contributes a better antioxidant effect of acidic polysaccharides than neutral polysaccharides [73,74,75]. It is also supposed that LBPs bind low molecular weight phenolic compounds during extraction from raw materials, which contributes to their antioxidant properties [76].

AA-2*β*G is also an important antioxidant compound of goji berries, which may share similar but distinct mechanistic properties with L-ascorbic acid [77].

Due to the structural diversity of bioactive compounds and their various mechanisms of antioxidant action, different methods have been used to determine the antioxidant activities of goji berry. Among others, the most frequently applied assays include 2,2-diphenyl-1-picrylhydrazyl(DPPH)radical,2,2′-azino-bis(3-ethylbenzothiazoline-6-sulphonic acid) (ABTS) radical cation scavenging activity, ferric reducing antioxidant power (FRAP), cupric ion reducing antioxidant capacity (CUPRAC), oxygen radical absorbance capacity (ORAC), and *β*-carotene bleaching inhibition.

In fact, in vitro studies have found a strong correlation between polysaccharides and phenolics with antioxidant activities [20,60,66,78], supporting that these compounds are the most significant contributors to the total antioxidant activities of goji berries [60]. On the other hand, the absence of a significant correlation between in vitro antioxidant activities (DPPH^•^, ABTS^•+^, and FRAP assays) and total carotenoids [60] could be explained by a lack of the enzyme which is capable of hydrolysing zeaxanthin esters to free zeaxanthin [79]. However, the abilities of goji berry extract to inhibit lipid peroxidation in the *β*-carotene-linoleic acid assay are linked to carotenoids content [17,25]. Namely, the presence of a long chain of conjugated double bonds in carotenoids was attributed to their more distinct effect in scavenging hydroxyl radicals compared to other bioactive compounds [74]. Furthermore, among carotenoids, there is evidence that zeaxanthin has the highest hydroxyl radical-scavenging activity hydroxyl radical-scavenging activities, followed by β-carotene, lycopene and lutein [80].

In addition to genetic differences, reports on the antioxidant activities of goji berries reflect differences in bioactive compounds affected by environmental conditions in various geographical regions, and extraction methods, among many others (Table 2).

## 4. Health Benefits and Side Effects of Goji Berry Consumption

### 4.1. Health Benefits of Goji Berry Consumption

The global popularization of goji berry and goji berry-based products is supported by scientific evidence on their health-promoting effects (Figure 1). In general, LBPs, zeaxanthin dipalmitate, vitamins, betaine, and mixed extracts have been attributed to the anti-aging, improving eyesight, anti-fatigue, and other beneficial effects of goji berry described in ancient herbals [7]. The main findings from human intervention studies demonstrating the health properties of goji berry, juice, or extracts are summarized in Table 3.

In 2008, the first randomized, double-blind, placebo-controlled clinical study was conducted, which reported the general effects of goji juice in healthy adults outside of China [82]. Participants in the intervention group consumed 120 mL/day of commercial goji juice (GoChi), standardized to contain LBP equivalent in at least 150 g of fresh fruit. Consistent with traditional use, the main beneficial effects observed in the intervention group after 14 days included increasing general well-being and improving neurological/psychologic performances and gastrointestinal functions. Other studies demonstrated that the 30-day daily consumption of GoChi led to the improvement of endogenous antioxidant enzyme activities, along with a decrease in malondialdehyde (MDA), an oxidative stress marker [83], and an increase in lymphocyte, IgG, and IL-2 levels in healthy subjects, without adverse effects [85]. In addition, it was found that GoChi consumption for 14 days increases metabolic rate and reduces the waist circumference in healthy subjects of both sexes [86].

As Yu, Wu, and Niu (2009) demonstrated, LBP extract exhibited favourable effects on plasma lipids and the risk of cardiovascular diseases to the elderly [84]. In addition, based on the observed hypoglycemic activities and increased HDL concentration, after three months of supplementation (300 mg/day), LBP has been considered as a prominent adjuvant therapy for patients with type 2 diabetes [89]. Although several mechanisms may account for these effects, there is a finding that the water goji berries extract exerted antioxidative and anti-inflammatory effects by controlling the expression of inflammatory mRNAs in overweight and hypercholesterolemia-suffering subjects [90]. In another study, the inclusion of 14 g of dried goji berry as part of a healthy diet after 45 days was associated with a significant reduction in transaminases and waist circumference, improved lipid profile, and oxidative stress parameters in patients with metabolic syndrome [91]. However, a meta-analysis of the effects of *L. barbarum* supplementation on cardiometabolic risk factors, which included 548 subjects, indicated only a favourable effect on glucose control, and a marginal reduction in total cholesterol and triglyceride levels, without any benefit to body weight and blood pressure [97]. Moreover, a single dose of 25 g of dried *L. barbarum* fruits did not influence the postprandial energy expenditure, plasma glucose, serum-free fatty acids, and triglycerides concentrations in healthy, overweight men [93].

According to Cheng et al. (2005), dietary supplementation with whole dried goji berry (15 g/day), estimated to contain 3 mg zeaxanthin, resulted in a 2.5-fold increase of plasma zeaxanthin. Based on this result, goji berry has been proposed as an inexpensive, effective, and safe food dietary strategy to increase plasma zeaxanthin concentration [81]. A recent study showed that 90 days of goji berry consumption was accompanied by an increase in macular pigment optical density, a marker of age-related macular degeneration in healthy, middle-aged adults [96]. Other studies demonstrated that dietary intervention with a proprietary milk-based formulation of goji berry (13.7 g/day), with enhancing zeaxanthin bioavailability [98], for 90 days, increases plasma zeaxanthin and improves antioxidant capacity and macular characteristics [87], as well as enhancing the immune system in healthy elderly subjects [88]. Moreover, the retinoprotective effects of 12-month goji berry supplementation were proven in patients with retinitis pigmentosa [92]. Thus, goji berry supplementation may represent a model of the successful integration of traditional Asian practices into Western medicine related to the prevention and treatment of retinal disorders [99].

Due to limited evidence on the health effects of whole dried fruits, considering possible synergism among bioactive compounds, Toh et al. (2021) investigated the effects of healthy dietary patterns, either with or without whole dried wolfberry (15 g/d), in middle-aged and older adults. They demonstrated that adherence to a healthy dietary pattern based on whole grains, fruits, non-starchy vegetables, and non-processed meats improves vascular tone, while incorporating goji berry into the diet further improves the blood lipid-lipoprotein profile, and may lower long-term cardiovascular risk [94]. In addition, they observed an inverse association between the changes in plasma zeaxanthin and plasma 8-iso-prostaglandin F2α. Therefore, goji berry within a healthy dietary pattern is proposed as a dietary strategy to attenuate lipid peroxidation among middle-aged and older adults, with a heightened risk of oxidative stress-induced age-related disorders [95].

Furthermore, a recent meta-analysis of goji berry-based RCTs indicated more pronounced effects of whole goji berry versus goji berry extract in improving the blood lipids and lipoproteins profile, supporting its incorporation into dietary patterns targeted at improving cardiovascular health [100].

Overall, findings from human interventional studies support the health benefits of goji berry consumption with the dosages used in traditional medicine, which range from 6–30 g of dried whole goji berry [12].

### 4.2. Side Effects of Goji Berry Consumption

Based on long-term traditional use over 2500 years, goji berry is now generally recognized as a non-toxic food [9,12]. Additionally, findings from dietary interventional studies indicate the safety of goji berry and its products when taken within traditionally established doses. However, some concerns about goji berries have been raised, especially regarding their consumption in large amounts. However, studies evaluating adequate dosage regimens, adverse reactions, and the long-term safety of goji berries and their products are scarce [12]. The oral administration of goji juice (GoChi) in rats demonstrated no toxicity, even at the maximum dose (10 mL/kg/day) [101]. However, high concentrations of goji juice should be carefully consumed, due to recently observed pro-oxidant effects and reducing the lifespan of the nematode *Caenorhabditis elegans*, a model organism for in vivo tests [102]. There are also reported cases of hepatotoxicity related to consuming goji berry [103,104].

Other risks related to goji berry include the presence of tropane alkaloids [9], chemical contaminants, such as pesticides and toxic elements [105,106], or some proteins that can cause allergic reactions in sensitive consumers [31]. However, it has been shown that the organic production system has a significant influence on the lower content of toxic elements [13], and the low contents of tropane alkaloids and pesticides, in general, have no toxicological relevance [9,106,107]. A recent study reported that the exposure of the dangerous effects of chemical residues is more likely from goji berries obtained from plantations than from goji berries from supermarkets, and that metal exposure is more dangerous than pesticide exposure [106].

Since the goji berry was introduced into the European market as a functional food and a nutraceutical a few years ago, its consumption has significantly increased. However, there is evidence from several countries that its consumption might lead to hypersensitive reactions. A 53-year-old male developed systemic photosensitivity due to the simultaneous prolonged use of goji berries and cat’s claw for 5 and 3 months, respectively. However, furthermore, the photo-provocations test revealed that photosensitivity is accompanied only by goji berry [108]. There is evidence that goji berries can cause allergic reactions in exposed and unexposed food-allergic individuals [109]. In addition, two cases of anaphylaxis were reported [110,111]. Cross-reactivity was demonstrated with peach, tomato, tobacco, a mixture of nuts, and *Artemisia* sp. pollen, while lipid transfer proteins (LTPs) are recognised as the major allergens involved in sensitisation and cross-reactivity [109,112]. Therefore, a high-cross reactivity with other frequently consumed foods could explain the high prevalence rate and the low frequency of clinical symptoms of goji berry allergies among European populations [31].

In addition, there is a risk of possible interactions between goji berry and medicines. There is evidence that the concomitant use of *Lycium* fruit tea, juice, and wine with oral anticoagulants, such as warfarin, might increase the risk of bleeding [113]. In a recent case report, Guzman et al. (2021) described the toxicity of the antiarrhythmic drug flecainidine in a 75-year-old female associated with goji berry tea consumption as a self-medication practice for the prevention of COVID-19 [114].

## 5. Processing of Goji Berries

### 5.1. Oven and Freeze-Drying Dehydration Techniques

Dehydration is one of the most commonly used processes for extending the shelf life of fruit, and it is based on the simultaneous transfer of heat and mass. Solar drying is a traditional drying method for goji berries. However, this type of drying and environmental conditions can cause a loss of fruit quality. In recent years, oven-air, freeze, or advanced drying techniques have been increasingly used for goji berries dehydration, thus preventing enzyme activity, reducing microbiological spoilage, and minimizing adverse reactions during storage [115]. However, drying goji berries is difficult due to the wax layer surrounding the peel of the berries, which impedes the diffusion of water from berries [115,116]. In addition, the wax layer of goji berries requires high temperatures and extended treatment, which can negatively affect the content of high-value compounds.

Thus, several studies have examined the possibility of using pre-treatment before drying, intending to speed up the drying process, reduce energy consumption, and improve the quality of dried products [115,116,117,118,119,120]. For example, abrasive pre-treatment in a motorized drum coated with sandpaper was used to carefully remove wax from the surface of goji berries, which shortened the drying time in the convective oven at 60 °C, preserved colour, increased antioxidant properties, and maintained the sugar content of berries [115,117,119]. In addition, washing and soaking goji berries in sodium carbonate solution before hot air drying increased the effective diffusivity and reduced the drying time; that is, it increased the content of bioactive compounds (total phenolics, flavonoids, carotenoids, and betaine) and improved the antioxidant activities of goji berries dried at 40 °C and 50 °C [120]. Moreover, sodium carbonate pre-treatment combined with hybrid drying techniques increased the pore size of goji berries, which accelerated the drying process and improved heat and mass transfer [121].

Pre-treatments, such as osmotic dehydration (60 min, 55 °C) and combined effect pulsed electric field and osmotic dehydration, have led to a decrease in drying time for the goji berry, maintaining its bright red colour, improving its texture, retaining its high antioxidant potential, and prolonging shelf life during the storage of dried goji berries [122,123]. The application of pulsed electric fields in pre-treatment affected the permeabilization of the tissue of goji berries, which increased the mass transfer during subsequent osmotic dehydration and air-drying [122]. The most conventional techniques use high drying temperatures, which most often impair the quality of the product (colour, taste, aroma, and texture), prevent rehydration, generate a Maillard reaction, and contribute to forming a hard coat around the berry [124]. However, although the texture of oven-dried goji berries is often disturbed, they are a good source of bioactive compounds such as phenolics, and show antioxidant activity [105,125].

On the other hand, quick-freezing using liquid nitrogen at −80 °C proved to be the most suitable freezing process for goji berries in comparison with other freezing temperatures at −60 and −100 °C, because, at this temperature, goji berries retain good sensory properties, have the lowest activity of polyphenol peroxidase, and more negligible damage of the internal structures of epidermal cells [126]. Furthermore, the advanced freeze-drying technique contributes to preserving goji berries´ bioactive compounds (phenolic acids, vitamin C, monoterpenes, and carotenoids) and nutrients. This results in high-quality, dried goji snacks which are light, crunchy, with original taste, preserved texture, and a healthy alternative to sweet snacks [124,127,128]. In addition, Song et al. (2018) reported that the application of advanced freeze-dried with instant controlled pressure drop drying for the dehydration of goji berries resulted in excellent crispness and texture, as well as high contents of total polysaccharides (139.8 g/kg) and carotenoids (2.43 g/kg) in dried products [121].

### 5.2. Advanced Techniques

In recent years, the effect of novel pre-treatments and the possibility of applying advanced techniques for drying goji berries have been increasingly examined. The recently developed cold plasma pre-treatment applied to goji berries has significantly reduced drying time, improved rehydration, and maintained the colour of berries [129]. After pre-treatment with cold plasma, the cell walls of the goji berries become thinner and more permeable, affecting the loss of bioactive compounds, probably degraded after interaction with charged particles generated by cold plasma during long treatment [129]. The electrohydrodynamic drying technique (EHD) has been successfully applied for the dehydration of goji berries, previously pre-treated with alkaline solution (KOH and NaOH), Na_2_CO_3_, sucrose ester, and ultrasonic. All applied pre-treatments accelerated drying, reduced drying time, affected energy savings, maintained good berry quality, and changed the surface microstructure of goji berries. However, only alkaline pre-treatment can be used as an optimal pre-treatment for the industrial application of goji berries dried using EHD [118].

Infrared drying has been successfully used for drying various fruit and vegetables, but this type of drying is not suitable for products sensitive to heat, so it is often combined with other drying methods [130]. Till now, far-infrared radiation heating assisted pulsed vacuum drying (FIR-PVD) [131], and a pilot-scale pulsed vacuum infrared drying system (PVID) [132] was used to dry goji berries. Both infrared-based drying techniques significantly reduced drying time and increased drying efficiency; that is, the goji berries had a more sustainable and attractive colour compared to hot air-dried berries. In addition, porous and fissured microstructures were observed in berries that were dried using FIR-PVD, and can potentially improve drying kinetics and rehydration [132]. Finally, the most favourable conditions for drying goji berries are a drying temperature (infrared heating) of 65 °C, a vacuum duration of 15 min, and atmospheric durations of 4 and 2 min [131,132].

A new technique of pulsed vacuum drying combined with carboxymethyl cellulose coating (optimal concentration was 2.0% *w/w*) allowed higher drying efficiency, less drying shrinkage, the preservation of colour, and an improvement of the quality of dried goji berries (total polysaccharides and phenolics) have been proposed as effective pre-treatment dehydration models for goji berries [133]). Carboxymethyl cellulose is a hydrocolloid that can form a flexible and transparent coating on the surface, limiting mass transfer resistance from inside the berry. At the same time, pressure pulsation affects the formation of a porous structure, which facilitates the transfer of mass and heat during drying [133]. Interestingly, Qi et al. (2021) suggest that low-intensity pulsed ultrasound-assisted vacuum drying at 50 °C with a pulsed ultrasound ratio of 10s:10s is a novel, promising drying technique for goji berries juice [134].

### 5.3. Encapsulation of Bioactive Compounds from Goji Berries

As previously mentioned, goji berries are a source of numerous bioactive components, such as polysaccharides, carotenoids, and phenolics. These molecules can be isolated from goji berries and further used in the food and pharmaceutical industries. The water-soluble bioactive components of goji berries are traditionally extracted using hot water as a solvent. In order to improve the extraction efficiency, numerous novel extraction methods have been developed, including ultrasound- or microwave-assisted aqueous extractions [135,136], as well as subcritical water extraction [137]. In addition to the extraction method, other factors may affect the extraction procedure, including the origin of fruits, selected solvent, extraction time, and temperature, etc. Several research groups have demonstrated that higher phenolic compounds can be extracted by using ethanol as a solvent rather than water [138]. The efficiency of other solvents for extraction has been studied, including ultrapure water, acetone, ethanol, and methanol in different concentrations and mixtures [139]. Zhou et al. (2020) have demonstrated that the extraction solvents (water, hydrochloric acid 0.4%, or sodium hydroxide 0.6% solution) used for extraction at different temperatures affect the structures of the extracted polysaccharides. Alkali extractions resulted in low galacturonic acid and higher protein content than acidic and water extractions. In particular, low-temperature alkaline extraction conditions have led to extensively branched rhamnogalacturonan I, whereas high-temperature acidic conditions provided homogalacturonan regions, and resulted in the removal of part of the side chain [140]. Ahmadi et al. (2022) have also reported that the highest galacturonic acid contents were obtained by hot acid extraction [141]. A similar effect was reported on the stability of black goji berry anthocyanins, under variable pH conditions [57]. Water or acidified solvents are predominantly used for anthocyanin extraction, and they also indicated that the storage stability of the crude black goji anthocyanin extract is high in acidic (pH of 3–4) and low in alkaline pH [57]. Therefore, if water is used as the extraction solvent, it is necessary to carefully investigate the influence of pH on the stability of the obtained extracts.

The extraction process can be optimised for high(est) yield, bioactivity, and/or resource efficiency, by varying the relevant parameters through an experimental design. The obtained, optimised extracts can be used for further processing. Box–Behnken, as a response surface design, and different factorial designs, have been reported to optimize the goji berries extraction procedures.

Jixian Zhang et al. (2021) reported that the polysaccharides of goji berries stabilized and maintained the morphology of selenium nanoparticles. Moreover, these polysaccharide-SeNPs possess structural stability during digestion, and can enhance the absorption and bioavailability of selenium [142]. In addition, the polysaccharides of goji berries in combination with some minerals or other macromolecules can easily lead to gelling, or help form gel network structures [143,144]. For example, the addition of Ca^2+^ ions plays a crucial role in forming the gel network structure in the polysaccharide of goji berries, which enables their broader application in the food industry [144]. On the other hand, goji polysaccharides support the formation of gels of heat-induced whey protein at different pHs, and encourage the development of new gel-type foods [143].

Carotenoids from goji berries are lipophilic and unstable molecules, easily degraded under the influence of light, heat, or oxygen. For this reason, they are often extracted from berries and incorporated into appropriate delivery systems, which improve their bioavailability. Till now, different types of oil, such as soybean, sunflower, palm, or cottonseed, have been applied as carriers of carotenoids separated from goji puree [145]. Moreover, the bioaccessibility of carotenoid esters, primarily zeaxanthin esters, is significantly increased when goji berries are mixed with O/W emulsion [146]. This also represents a new food model for increasing the bioaccessibility of carotenoids from different fruits. According to recent studies, carotenoid esters extracted or separated from goji berries have been incorporated into nanoemulsion [147], or nanocarriers formed by complex coacervation between gelatin and sodium carboxymethyl cellulose [148]. De Campo et al. (2018) have exploited the use of cactus cladode mucilage (*Opuntia monacantha*, (Willd.) Haw., Cactaceae) as an encapsulation agent for zeaxanthin. Due to its lipophilic properties, the encapsulation of zeaxanthin by the mucilage polysaccharides may provide improved solubility in water. Furthermore, it has been demonstrated that the encapsulation provided improved stability against various agents [149]. Hempel et al. (2017) have studied the effect of the ripening of goji berries on the conversion of carotenoids from being bound to chloroplastidal thylakoids, to their accumulation in the nano-scale liquid-crystalline state, predominantly in the form of zeaxanthin dipalmitate [79].

Lipid-based nanoparticles were derived from goji berries, specifically from the lipid contents extracted from goji berries [150]. The lipidomic analysis of the obtained nanoparticles was performed, as well as the analysis of the bioactive flavone. Vitexin-2-O-rhamnoside was detected in the lipid nanoparticles. Experiments have indicated the capacity of goji berries’ lipid nanoparticles to inhibit the secretion of the main pro-inflammatory cytokines, and to regulate the expression of anti-inflammatory factors. It has further been demonstrated that the obtained nanoparticles can relieve ulcerative colitis symptoms.

Polyphenols from goji berries’ leaves have been encapsulated in liposomes to improve their delivery in terms of sustaining their release, and avoiding the burst effect compared to the dissolution of the free extract [151]. The obtained liposomes have successfully served as carriers for polyphenols and demonstrated a cytoprotective effect on L-929 mouse fibroblasts cells.

Finally, the whole aqueous extracts of goji berries encapsulated in maltodextrin can be used as potentially prebiotic food additives, because they have been shown to support growth and viability, and stimulate the proliferation of probiotic strains of bacteria, such as Bifidobacterium and Lactobacillus, in simulated gastrointestinal conditions [152]. The same research group [153] has also demonstrated that the aqueous extracts of goji berries, where the encapsulation was performed with minimal maltodextrin content and high polyphenols content, had high antioxidant and antimicrobial activity. Such products could be used for the preservation of food or plant protection.

## 6. Goji Berry as Source of Functional Ingredients in Different Food Products

**Figure 2:**
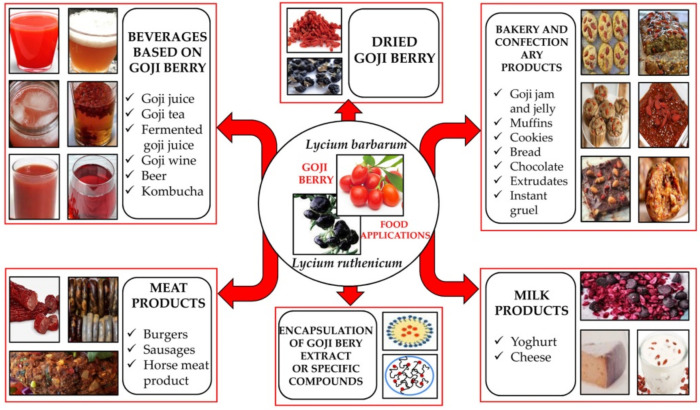
Goji berry-based functional food products.

Recently, goji berries have become increasingly used as a raw material for the production of specific beverages, or incorporated into various food products, such as bakery, confectionery, meat, and milk products, contributing to their nutritional, health-promoting, and sensory properties (Figure 2) [18,20]. The applications of goji berries as functional ingredients in the formulation of different food products are reviewed and shown in Table 4.

### 6.1. Goji Berry as a Raw Material or a Functional Ingredient in the Production of Beverages

Goji berries (red and black) were used for goji juice production [102,154], goji tea [70], and specific fermented beverages such as fermented goji juice [155,156,157,158], goji wine [159,160], and kombucha beverages [161]. Fermented goji beverages had high TPC, TFC, and TAcy content, as well as good in vitro antioxidant properties evaluated using DPPH^•^, ABTS^•+^, FRAP, CUPRAC, and ORAC assays [155,156,159,161]. More precisely, bioactive compounds such as phenolic acids (*p*-hydroxybenzoic, *p*-coumaric, and ferulic acid), rutin, and various volatile compounds were predominantly identified in fermented beverages [156,158,162]. In addition, goji juice fermented by probiotics (*Lactobacillus* and/or *Streptococcus* sp.) showed high cellular antioxidant activity (HepG2 cells) [155], improved function against ulcerative colitis, decreased intestinal permeability, and modulated gut microbiota [157]. Moreover, fermented goji beverages generally had moderate colour intensity, a specific odour/flavour (fruity, honey, floral and vanilla) related to volatile compounds, balanced taste (slightly sour and sweet), and good acceptability by consumers [156,158,159].

Goji berries were also successfully used as a functional ingredient in different stages of the production of amber ale beer [162]. Beer with goji berries had increased contents of total phenolics, some individually phenolic compounds (rutin, *p*-coumaric, and ferulic acid) and AA-2*β*G, as well as good antioxidant potential. In addition, these beers had good sensory acceptability with specific sensations of odour and taste, such as red fruit, honey, caramel, coffee, hay, smoke, and a balanced ratio of bitterness and sourness [162]. Finally, black goji extract can be used as a functional ingredient and natural colour in many food products [163]. This extract predominantly contains petunidin derivatives, primarily *cis* and *trans* isomers of petunidin-3-*p*-coumaroyl-rutinoside-5-*O*-glucoside, responsible for colour retention and improvement intensity, and the stability of colour in wide pH ranges [163].

### 6.2. Goji Berries as Functional Ingredients in Meat Products

Goji extract has been successfully incorporated in products of minced catfish [164] and horse meat [165,166] to improve sensory properties and oxidative stability during storage. Moreover, chitosan/goji extract can be used as a biopreservative and antilisterial agent when mixed with minced catfish [164].

The addition of goji berries or extract in sausages effectively suppressed lipolysis and protein/lipid oxidation, reduced microbial count during storage, and preserved the bright red colour, fresh aroma, and taste of sausages [167,168]. Furthermore, beef burgers with different shares of goji puree (0%, 2.5%, and 5%) had increased total phenolic content, decreased lipid peroxidation, and good antioxidant properties, as well as a pleasing odour, taste, flavour and texture for various groups of consumers (young, adult, and elderly) [169]. Interestingly, the meat of rabbits fed with goji berries as a dietary supplement had increased total phenolic content, decreased oxidation and unchanged colour, water-holding capacity, and muscle tenderness [170,171]. Moreover, meatballs made from the meat of rabbit fed with goji berry had a high sensory score and were more acceptable in terms of colour, juiciness, taste, and overall liking [171].

### 6.3. Goji Berries as Functional Ingredients in Confectionery and Bakery Products

Goji berry sweet products, such as jam or jelly, had good antioxidant potential and high scores for colour, consistency, flavour, and sweetness. However, these products had lower sensory scores for sourness and aftertaste [172]. On the other hand, different confectionery and bakery products can be fortified with various shares of goji berries to improve their functional, sensory, or texture properties. For example, rice extrudates and instant gruels with an increasing share of dry goji berry in mixture had increased content of total phenolics, some individually phenolic acids, rutin, zeaxanthin dipalmitate, and AA-2βG, as well as higher antioxidant properties [173,174].

In addition, muffins and cookies enriched with different shares of goji berries powder or by-products had increased total phenolic, insoluble, and soluble fibre contents, and good sensory properties (sourness, slightly sweet and specific flavour). They darkened the colour of the extrudates via the Maillard reaction [175,176]. However, muffins and cookies enriched with goji berry by-products had decreased firmness and hardness/fracturability, respectively [175]. Furthermore, gluten-free bread, progressively enriched with goji berries in the range of 0% to 15%, had decreased bread volume, hardness, lightness of bread crumbs, increased redness, elasticity, and cohesion of bread crumb [177]. The addition of goji berries (9% *w/w*) in prebiotic chocolates has changed the perception of most aroma, flavour, and texture attributes; that is, it increased and improved the bitter taste, bitter aftertaste, astringency, adherence, grittiness, hardness, and aroma of the goji berry [178]. More precisely, chocolate with goji berries had consumer acceptance scores above six on a 9-point scale.

### 6.4. Goji Berries as Functional Ingredients in Milk Products

Incorporating goji berries or extract into milk products such as probiotic yogurt [179,180,181] and cheese [182] contributes to an increase in the TPC and antioxidant activities of these products. Goji berries also improved the viability of lactic acid bacteria in yoghurt during storage [180]. Furthermore, cheese with goji extracts showed decreased inhibitory activity in the angiotensin-converting enzyme (ACE). However, cheese enriched with goji extract/fish collagen had the highest production of peptides after the 14th and 28th days of storage, which could potentially have anti-ACE activity [182]. Finally, in the study of Rotar et al. (2015), a sensory analysis showed that consumers preferred yogurt with 7% goji berries, with a score of 8.21 points on the hedonic scale [180].

**Table 4:** An overview in the functional food products development using goji berries or goji berry products.

Products	Goji Berries	Microbial Species Involved in Fermentation	Main Observations		Specific Note	Reference
			Functional Properties	Sensory and Texture Properties		
**BEVERAGES**						
Kombucha beverages	Dried goji berries: 1. Red goji berry (*Lycium barbarum* L.) 2. Black goji berry (*Lycium ruthenicum* Murr.)	Kombucha culture: Symbiosis of acetic acid bacteria and yeast species	1. High TPC 2. High antioxidant properties (DPPH^•^, FRAP and CUPRAC)	1. Decrease colour intensity due to microbial transformation of phenolics (high score) 2. Odour highly acceptable(smell from fruity to acetic acid) 3. Taste (fruity, sour and sparkling flavour)	Increased TPC and antioxidant properties (except DPPH^•^) after in vitro digestion	[161]
Amber ale beer	Dry goji berries	*Saccharomyces cerevisiae* yeast	1. High TPC 2. High rutin, *p*-coumaric and ferulic acid content 3. High content of AA-2*β*G 4. High antioxidant activity (TEAC and ORAC)	Hedonic score: Appreciation 1. Lower intensity 2. High colour intensity 3. Odour (red fruit, grainy, honey, caramel, coffee, hay-like and smoky) 4. Taste (bitterness, sourness, red fruit-, caramel-, coffee- and grainy-like)	Goji berries were added to ale type beer at different stages of the production process	[162]
Fermented goji juice	Goji berries extract	*Lactobacillus plantarum* RV21797	1. High TPC, TFC and TAcy 2. Expressed in vitro antioxidant properties (DPPH^•^, ABTS^•+^, FRAP and ORAC) 3. High cellular antioxidant activity (HepG2 Cells)	/	/	[155]
Fermented goji juice	Dried goji berries (soaked goji berries)	*Bacillus velezensis*, *Bacillus licheniformis*, *Lactobacillus reuteri, L. rhamnosus* and *L. plantarum*	1. High TPC and TFC 2. High *p*-hydroxybenzoic acid, *p*-coumaric acid and rutin content 3. High individually volatile compounds content 4. High antioxidant activity (DPPH^•^ and hydroxyl radical)	1. Colour is moderate, very good 2. Flavour (aroma of goji juice is pure and has no odour) 3. Taste (slightly acidic or sweet) 4. Acceptability (very like)	/	[157]
Goji berry tea	Dried goji berries: 1. Red goji berry (*Lycium barbarum* L.) 2. Black goji berry (*Lycium ruthenicum*)	/	1. High TPC 2. High LBP content 3. High antioxidant activity (DPPH^•^, ABTS^•+^ and FRAP)	1. Colour of red and black goji berry tea were light yellow and purple, respectively, and then colour gradually changed to darker with the increase of time and temperature of soaking	This study monitored the effects of various temperatures and times of soak on antioxidant properties of specific goji berry tea.	[70]
Fermented goji juice by probiotics	Dried goji berries (soaked goji berries)	*Lactobacillus plantarum*, *L. reuteri* and *Streptococcus thermophilus*	1. Decreases the levels of pro-inflammatory cytokines and total superoxide dismutase in serum and colon 2. Increased the levels of anti-inflammatory cytokines, myeloperoxidase and glutathione peroxidase 3. Decreases intestinal permeability 4. Modulate gut microbiota	/	Probiotics fermentation of goji berry juice contributing to enhanced the anti-ulcerative colitis function.	[157]
Fermented goji juice	Fully ripe and frozen goji berries (goji purre crashed with pectinase)	*Lactiplantibacillus plantarum, Lactobacillus acidophilus, L. helveticus, Fructobacillus fructosus, Weissella cibaria*	1. Highly individually volatile compounds content (93 volatile compounds and seven non-volatile organic acids)	1. Juices fermented with L. *plantarum* or L. *acidophilus* were described with ‘honey’, ‘wild jujube’ odours and ‘sour’ taste 2. Juices with L. *helveticus* were described with ‘goji berry’, ‘floral’ and sweetness 3. Juices with F. *fructosus* or W. *cibaria* were described with ‘vinegar’ and sweetness	L. *helveticus* 6024 is the most active strain able to retain or liberated the key compounds positively associated with ‘goji berry’ note.	[158]
Goji juice and goji capsules	Goji berries	/	1. Source of nutritional and mineral elements	/	Goji capsules contained higher concentration of all individually minerals compared to goji juice samples.	[154]
Goji wine	Dried goji berries (goji puree)	*Saccharomyces cerevisiae*	1. High TPC and TFC 2. High LBPs content 3. High individually volatile compounds content 4. High antioxidant activity (DPPH^•^ and ABTS^•+^)	1. Flavour (woody, vanillia and clove aroma), aroma related with compounds such as *cis*- and *trans*-whisky lactone, vanillin, eugenol, isoeugenol, and 4-vinylguaiacol 2. The highest score in olfactory and gustative attributes	This study monitored the effects of various oak matrices (medium toast barrel, medium toast shavings, non-toast chips, light toast chips, medium toast chips, and heavy toast chips) on the volatiles and antioxidant activity in Goji wine. Thus, Goji wine treated with oak shavings had the highest antioxidant activity, phenolics and flavonoids content	[159]
Goji berry juice	Dehydrated goji berries	/	1. High protocatehuic acid, vanillic acid, *p*-coumaric acid, catechin and rutin content 2. Goji juice caused toxicity and reduced the lifespan of *Caenorhabditis elegans* 3. Goji juice increased lipofuscin, glucose levels, number of apoptotic bodies and lipase activity	/	High concentration of goji juice showed toxic effects and promoted premature aging in *C. elegans*. Thus, goji juice should be carefully consumed until further studies are conducted.	[102]
Black goji extract as source of natural colour	Dried black goji berries	/	1. High content of petunidin derivatives, primarily *cis* and *trans* isomers of petunidin-3-*p*-coumaroyl-rutinoside-5-*O*-glucoside	Acylated petunidin anthocyanins are responsible for colour retention and improvement of colour intensity and stability.	Black goji anthocyanins produced various colour shades in broad ranges of pH.	[163]
Goji wine	Dried goji berries (mixed with water and decomposed with pectolase)	*Saccharomyces cerevisiae*	/	/	Ethyl carbamate was formed during the fermentation and storage processes of goji wine.	[160]
**MEAT PRODUCTS**						
Minced catfish	Goji berry extract	/	/	High score for odour, texture, colour and overall quality of catfish minced blended with chitosan/goji berry extract, immediately after mixing and after 14 days of storage.	Chitosan/goji berry extract can be used as a biopreservative and anti-listerial agents (prevents the growth of *Listeria monocytogenes*), and also enhanced sensory properties and storage stability, when is mixed with catfish minced.	[164]
Beef burgers	Goji puree (0%; 2.5%; and 5%)	/	Burgers with goji: 1. Increased TPC 2. Improved antioxidant properties (DPPH^•^, ABTS^•+^ and ORAC) 3. Decreased lipid peroxidation	Burgers had acceptable appearance, odour, taste, flavour and texture for all groups of consumers (young, adult and elderly)	Burgers with goji had significantly higher TPC and antioxidant properties after in vitro digestion	[169]
Cooked sausages	Dried goji berries (0.5% and 1%)	/	/	The addition of 0.5% goji berries had the highest contribution to the preservation of bright red colour, fresh aroma and taste of functional cooked sausages	The addition of 0.5% and 1% of goji berries effectively inhibited protein oxidation, lipolysis, and lipid oxidation in functional cooked sausages	[167]
Rabbit meat	Rabbit feed was supplemented with 3% goji berries	/	/	Consumers gave a higher score for meatballs produced of meat of rabbits which were fed with goji berries dietary supplementation. These samples had more acceptable colour, juiciness, taste and overall liking.	Meat obtained of rabbits fed with goji berries dietary supplementation had reduced TBARS values and significant impact on *Lactobacillus* spp. prevalence.	[170]
Smoked common carp sausages	Goji berry extracts (1% and 2%)	/	/	1. Sausages with goji berry extract had partial redness colour 2. The highest score of aroma and colour had sausages with 1% goji extract.	Sausages supplemented with goji extracts had decreased TBA values, TVB-N contents and total aerobic mesophilic bacteria during storage, in comparison to control sample (without goji).	[168]
Cooked and smoked horse meat product	Goji berry extract and goji berry extract/buckwheat flour	/	/	A high score for appearance, shear, colour, taste, odour and consistency were evaluated for a horsemeat product enriched with goji extract or goji/buckwheat mixture.	/	[165]
Rabbit meat	Rabbit feed was supplemented with 1% and 3% goji berries	/	Meat obtained of rabbits fed with goji berries dietary supplementation: 1. Increased TPC 2. Improved antioxidant properties (ORAC)	Goji berries dietary supplementation did not affect the colour, water holding capacity and tenderness of rabbit meat muscle.	Meat obtained of rabbits which were fed with 3% goji berries dietary supplementation showed an increase in oxidative stability.	[171]
Kazakh horse-meat product	Goji berry extract (0.5% and 1%)	/		A high score for surface colour, smell and taste were evaluated for a horsemeat product enriched with 0.5% and 1% goji extract.	Horse meat products with 1.0% of goji extract had improved oxidative stability. On the other hand, adding of goji berries had destructive effect on most meat fiber.	[166]
**BAKERY AND CONFECTIONERY PRODUCTS**						
Goji jam and jelly	Dehydrated goji berries	/	1. High antioxidant activity (DPPH^•^)	Both goji products had high score for colour, appearance, consistency, flavour and sweet taste, however, for sour taste and aftertaste products had lower scores.	/	[172]
Muffins and spritz cookies	Whole goji berries (0% and 10%) Goji powder (0%, 3%, 5% and 10%)	/	/	1. Pastry products with goji berries had a sour, slightly sweet and specific flavour. 2. Consumer’s preferred muffins with 10% whole goji and cookies with 5% goji powder.	/	[176]
Muffins and cookies	Goji berry by-products (0%, 10%, 20%, 30% and 40%)	/	Bakery products enriched with goji by-products: 1. Increased TPC 2. Increased insoluble and soluble fibre	1. Increased goji by-products level decreased muffin firmness, that is, hardness and fracturability of cookies. 2. Muffins with 20% of goji by-products and cookies with 10% of goji by-products had the best sensory properties.	/	[175]
Prebiotic white chocolate	Dried goji berries (9% *w/w*)	/	/	1. According to quantitative descriptive analysis, adding goji berries in chocolate reduced the perception of most aroma and flavour attributes, and improved the bitter taste, bitter aftertaste, astringency, and most of the texture attributes 2. Increased adherence, grittiness, hardness, astringency and goji berry aroma in comparison with control sample. 3. Chocolates enriched with goji berries had acceptance scores above 6 on a 9-point scale.	/	[178]
Rice flour based extrudates	Dry goji berries (0%, 13%, 23% and 28.5%)	/	Increasing goji berry level in rice flour based extrudates resulted in: 1. Increased TPC 2. Increased antioxidant activity (DPPH^•^ and ABTS^•+^) 3. Increased rutin, zeaxanthin dipalmitate and AA-2βG content (content of listed compounds were higher in samples before extrusion)	/	/	[173]
Instant gruels	Dry goji berries (1%, 3% and 5%)	/	Increasing goji berry level in instant corn gruels resulted in: 1. Increased TPC 2. Increased antioxidant activity (TEAC and TLC-DPPH^•^) 3. Increased protocatehuic, 4-OH-benzoic, *p*-coumaric, ferulic, isoferulic and salicylic acid content	/	This study also monitored time (10 and 15 min) and rotation speed of the extruder screw (80 rpm, 100 rpm and 120 rpm)	[174]
Gluten-free bread	Dried goji berries (0%, 3%, 6%, 9%, 12% and 15%)	/	/	1. Goji addition in bread, range 3–12% had no significant influence on bread volume, while addition of 15% caused reduction in volume 2. Goji addition in bread reduced lightness and increased redness of bread crumb 3. Increasing of goji in bread influenced on decreased hardness of bread crumb and increased elasticity 4. Increasing goji content from 3% to 6% in bread influenced on increased cohesion of the crumb	/	[177]
**MILK PRODUCTS**						
Yoghurt	Dried goji berries (aqueous/ethanolic extract) (0.05%, 0.1% and 0.15% *w/v*)	Commercial yoghurt culture (yo-FAST-88), Hansen, Denmark	Increasing goji extract level in yoghurt resulted in: 1. Increased TPC 2. Increased antioxidant activity (DPPH^•^)	Increasing goji extract level in yogurt decreased consumer acceptability, with the same trend at the 1st day and after 20 days.	/	[179]
Yoghurt	Goji berries with/without honey (0%, 3%, 5% and 7%)	Starter mezophylic culture Lyofast Y450B (*Streptococcus thermophilus* and *Lactobacillus delbrueckii* subsp. *bulgaricus* (ratio 1:1))	/	Consumers preferred yoghurt with 7% goji berries (8.21 points on hedonic scale)	Goji berries maintained viability of lactic acid bacteria in yoghurt during storage	[180]
Yoghurt	Dry and ground goji berries (2%, 4% and 6%)	*Lactobacillus delbreukii* ssp. *bulgaricus*, *Streptococcus* *thermophilus*	Increasing goji extract level in yoghurt resulted in: 1. Increased TPC 2. Increased antioxidant activity (DPPH^•^)	/	Total phenolic content and antioxidant activity of yogurt enriched with goji berries is continuously reduced after 3th, 7th and 14th days of storage.	[181]
Cheese	1. Dried goji berries (3% water extract) 2. Goji extract/fish collagen	Lactic acid bacteria	1. Cheese with goji extract showed decreased ACE inhibitory activity 2. Cheese enriched with goji extract and fish collagen had the most enhanced peptides production after 14th and 28th days of storage, and potential anti-ACE activity.	/	/	[182]

“/”—not analysed; TPC—total phenolic content; TFC—total flavonoid content; TAcy—total anthocyanin content; LBPs—*L. barbarum* polysaccarides; AA-2βG: 2-*O*-β-d-glucopyranosyl-L-ascorbic acid; DPPH 2,2′-diphenyl-1-picrylhydrazyl radical scavenging activity; FRAP—ferric reducing antioxidant power; CUPRAC-cupric ion reducing antioxidant capacity; TEAC-trolox equivalent antioxidant capacity; ORAC—oxygen radical antioxidant capacity; ACE—angiotensin-converting enzyme; TBA—thiobarbituric acid; TVB-N: total volatile basic nitrogen.

## 7. Conclusions and Future Directions

Increasing scientific evidence on the health-promoting effects of goji berries has increased interest in the possible application of goji berries or their extracts as raw materials or functional additives in various food products. Several important conclusions regarding goji berries valorisation in the functional food industry can be drawn from previous research studies: (1) Goji berries are sensitive and easily susceptible to spoilage, which requires new postharvest and storage investigation to prolong the stability of fresh goji berries; (2) Dried goji berries can be found most often in the market, but in the future, more attention should be focused on applying modern drying technologies with appropriate pre-treatments to produce dried goji berries with better nutritional value and sensory properties; (3) Goji berry products or products fortified with goji berry have improved nutritional, functional, and sensory properties; (4) Sensory and texture properties generally depend on the share of goji berry added in the final products; (5) Consumption of highly concentrated goji products such as juice can potentially have an adverse effect and require additional research in the future; (6) New micro- and nanoencapsulation models should be investigated in the future, to protect sensitive compounds from goji berries during processing, and ensure their better absorption during digestion; (7) Further research should also examine the effect of the food matrix on the phenolic profile and the functional properties of incorporated goji berries; (8) During the processing of goji berries, significant quantities of their by-products are formed. Thus, in the future, attention should be paid to the valorisation of goji by-products and their application in the food industry; (9) It has been shown that goji berries’ addition to feeding improves rabbit meat quality; this should also be confirmed with other animals.

Finally, the efficacy and safety of goji berry-based food products and nutraceuticals need to be proven through well-designed clinical trials. Moreover, the possible interactions of goji berry with conventional medications and natural health products need investigation in more detail.

## Figures and Tables

**Table 1:** The total content of phenolics, flavonoids, carotenoids, and polysaccharides in goji berries.

TPC (mg/g)	TFC (mg/g)	TCC (mg/g)	LBP (mg/g)	Reference
*L. barbarum*				
2.56–2.82	-	5.7	-	[8]
11.6–15.7	-	-	-	[16]
4.0–13.0	-	4.0–9.5	-	[21]
7.17	2.37	0.43	-	[24]
1.62	2.14	0.42	-	[25]
3.89–8.20	-	2.9	-	[26]
0.71–2.94	-	-	-	[27]
0.25–1.93	-	0.66–4.13	-	[28]
7.6	-	-	-	[33]
6.9–8.25	3.18–6.14	12.93–25.35	23.62–42.45	[38]
			16–48	[47]
30.3–73.4	38.5–54.7	3.64–11.33	55.9–62.7	[60]
6.9–10.9	-	-	-	[61]
2.17–4.48	2.67–3.16	0.21–0.23	-	[63]
8.36–14.13	-	0.42–1.01	-	[64]
8.16–9.04	1.78–2.63	-	-	[65]
3.45–3.47	2.20–2.23	-	-	[66]
0.01–5.47	-	-	-	[67]
*L. ruthenicum*				
2.96	0.27	nd ^1^	-	[25]
26.9	36.1	0.40	56.1 ^2^	[60]
7.26–9.01	9.77–12.32	0.001–0.003	-	[63]
3.44–6.45	5.66–11.16	-	-	[66]
21.14–28.52	1.23–1.38	-	-	[68]
49.07	-	-	-	[69]

^1^ nd—not detected; TPC—total phenolic content; TFC—total flavonoid content; TCC—total carotenoid content; LBP—*L. barbarum* polysaccharides content; ^2^ LRP—*L. ruthenicum* polysaccharides content.

**Table 2:** Antioxidant properties of goji berries.

Sample Origin	Extraction Solvent	DPPH•	ABTS^•+^	FRAP	Reference
*L. barbarum*					
China	ethanol (60%, *v/v*)	44.63–47.63%	-	0.15–0.17 µmol Fe^+2^/g	[66]
	methanol (80%, *v/v*)	35.88–85.46 µmol TE/g fw	59.3–95.6 µmol TE/g fw	57.7–92.5 µmol TE/g fw	[60]
	Acetone/water/acetic acid (70:29.5:0.5)	16.07–17.47 µmol TE/g	53.92–64.38 µmol TE/g	26.39–46.51 mmol Fe^+2^/g	[63]
Greece	water	1.29–3.00 mg/mL (IC50)	0.42–1.10 mg/mL (IC50)	-	[22]
	water	0.83–1.15 mg/mL (IC50)	0.19–0.4 mg/mL (IC50)	-	[61]
Italy	methanol: water acidified with HCL	-	-	18.00–20.89 µmol Fe^+2^/g fw	[8]
North Macedonia	water	1.51–6.25 mg/g dw	1.94–9.93 mg /g dw	-	[26]
Poland	methanol (80%, *v/v*) + 1% HCl	-	16.0–68.3 µmol TE/g	14.4–63.0 µmol TE/g	[28]
Portugal	methanol (80%, *v/v*)	6.25 mg/mL (EC50)	-	-	[20]
Romania	methanol (70%, *v/v*)	8.79–9.35 mg TE/g	24.86–25.12 mg TE/g	16.91–19.52 mg TE/g	[16]
Serbia	methanol (80%, *v/v*)	4.52 µmol TE/g fw	0.12 µmol TE/g fw	5.32 µmol TE/g fw	[25]
Switzerland	methanol		6.94–13.22 µmol TE/g dw	-	[27]
Turkey	water	22.64 mg/mL (EC50)	-	2.93 mM Fe^+2^	[65]
	methanol (80%, *v/v*)	18.19 mg/mL (EC50)	-	2.62 mM Fe^+2^	
*L. ruthenicum*					
China	ethanol (85%, *v/v*)	315.7–460.5 µmol TE/g dw	327.8–485.6 µmol TE/g dw	377.0–539.4 µmol TE/g dw	[70]
	ethanol (60%, *v/v*)	63.09–85.15%	-	0.55–0.62 µmol Fe^+2^/g	[66]
	methanol (80%, *v/v*)	49.65 µmol TE/g fw	47.8 µmol TE/g fw	56.3 µmol TE/g fw	[60]
	acetone/water/ acetic acid (70:29.5:0.5)	32.29–35.86 µmol TE/g	147.00–180.03 µmol TE/g	278.21–363.46 mmol Fe^+2^/g	[63]
Serbia	methanol (80%, *v/v*)	10.22 µmol TE/g fw	0.28 µmol TE/g fw	19.43 µmol TE/g fw	[25]

TE—Trolox equivalent; fw—fresh weight; dw—dry weight.

**Table 3:** Clinical studies on the effects of goji berries and their products.

Study Design	Study Population	Number	Intervention (Dose)	Main Outcomes	Reference
Single-blinded, placebo-controlled, parallel design study	Healthy adults	27	28 days (15 g/d wolfberries-estimated to provide ~3 mg/d esterified zeaxanthin)	plasma zeaxanthin increased 2.5-fold	[81]
Double-blinded, placebo-controlled RCT	Healthy adults	34	14 days (120 mL/d LBP standardized juice—equivalent at least 150 g of fresh fruit)	↑ subjective feelings of general well-being, neurologic/psychologic performance and gastrointestinal functions	[82]
Double-blinded, placebo-controlled RCT	Healthy adults	39	30 days (120 mL/d LBP-standardized juice)	↑ SOD, GSH-Px ↓ lipid peroxidation (MDA)	[83]
Parallel design intervention study	Healthy elderly subjects	177	3 months (LBPs)	↓ plasma triglycerides, total cholesterol, and LDL cholesterol ↑ HDL cholesterol	[84]
Double-blinded, placebo-controlled RCT	Older healthy adults	60	30 days (120 mL/d LBP standardized juice—equivalent at least 150 g of fresh fruit)	↑ several immunological responses and subjective feelings of general well-being	[85]
Double-blinded, placebo-controlled RCT	Healthy adults	28	14 days (120 mL/d LBP standardized juice—equivalent at least 150 g of fresh fruit)	↓ waist circumference	[86]
Double-blinded, placebo-controlled RCT	Healthy elderly subjects	150	90 days (13.7 g/d milk-based formulation of goji berry, LWB)	↑ plasma zeaxanthin and antioxidant levels protects from hypopigmentation and soft drusen accumulation in the macula of elderly subjects	[87]
Double-blinded, placebo-controlled RCT	Healthy elderly subjects	150	90 days (13.7 g/d milk-based formulation of goji berry, LWB)	↑ postvaccination serum influenza-specific immunoglobulin G levels and seroconversion rate	[88]
Double-blinded, placebo-controlled RCT	Type 2 diabetes patients	67	90 days (300 mg LBPs/d)	↓ glucose and ↑ insulinogenic index ↑ HDL cholesterol	[89]
Double-blinded, placebo-controlled RCT	Healthy overweight and mild hypercholesterolemic subjects	53	8 weaks (80 mL/pouch-contained 13.5 g of WBE)	anti-oxidative and anti-inflammatory effects by modulating mRNA expression	[90]
Parallel design RCT	Metabolic syndrome patients	50	45 days (14 g dried goji berry with healthy dietary pattern)	↓ transaminases and waist circumference ↑ serum antioxidant capacity and GSH ↓ lipid peroxidation	[91]
Double-blinded, placebo-controlled RCT	Retinitis pigmentosa (RP) patients	42	12 months (10 g of LB granules/d, estimated to provide 0.175 g LBPs)	LB supplement provides a neuroprotective effect for the retina and could help delay or minimize cone degeneration in RP	[92]
Double-blind crossover RCT	Healthy, overweight men	17	25 g of dried LB fruit	≠ postprandial energy expenditure, substrate oxidation, and markers for lipid and glucose metabolism	[93]
Parallel design RCT	Middle-aged and older adults	40	16 weak (15 g/d whole, dried wolfberry with healthy dietary pattern)	improves vascular tone ↓ lipid peroxidation (8-iso-prostaglandin F2α)	[94,95]
Parallel design RCT	Healthy, middle-aged subjects	27	3 months (25 g of whole goji berries or supplements of lutein and zeaxhantin)	↑ macular pigment optical density	[96]

RCT—randomized control trial; RP—retinitis pigmentosa; LB—*L.barbarum*; LBPs—*L.barbarum* polysaccharides; LWB—Lacto-Wolfberry; SOD—superoxide dismutase; GSH-Px—glutathione peroxidase; MDA—malondialdehyde; LDL—low density lipoprotein; HDL—high density lipoprotein; WBE—aqueous extract of wolfberry; GSH—glutathione.

## Data Availability

Data are contained within the article.
